# Testing the efficacy of INtegral Cognitive REMediation (INCREM) in major depressive disorder: study protocol for a randomized clinical trial

**DOI:** 10.1186/s12888-019-2117-4

**Published:** 2019-05-06

**Authors:** Muriel Vicent-Gil, Beatriz Raventós, Eduardo D. Marín-Martínez, Sara González-Simarro, Anabel Martínez-Arán, Caterina del Mar Bonnin, Joan Trujols, Josefina Pérez-Blanco, Javier de Diego-Adeliño, Dolors Puigdemont, Maria Serra-Blasco, Narcís Cardoner, Maria J. Portella

**Affiliations:** 1 Department of Psychiatry, Hospital de la Santa Creu i Sant Pau, Centro de Investigación Biomédica en Red de Salud Mental (CIBERSAM), Institut d’Investigació Biomèdica Sant Pau (IBB-Sant Pau), Universitat Autònoma de Barcelona (UAB), Sant Antoni Ma Claret 167, 08025 Barcelona, Catalonia Spain; 2 Bipolar and Depressive Disorders Unit, Institute of Neurosciences, Hospital Clínic de Barcelona, Centro de Investigación Biomédica en Red de Salud Mental (CIBERSAM), IDIBAPS, University of Barcelona (UB), Barcelona, Catalonia Spain; 3 Mental Health Unit, Hospital Universitari Parc Taulí, Centro de Investigación Biomédica en Red de Salud Mental (CIBERSAM), Institut d’Investigació i Innovació Parc Taulí (I3PT), Universitat Autònoma de Barcelona (UAB), Sabadell, Barcelona, Catalonia Spain

**Keywords:** Cognitive remediation, Depression, Functional remediation, Computerized cognitive training, Clinical trial

## Abstract

**Background:**

Given the limitation of pharmacological treatments to treat cognitive symptoms in patients with Major Depressive Disorder (MDD), cognitive remediation programs has been proposed as a possible procognitive intervention but findings are not conclusive. This study investigates the efficacy of an INtegral Cognitive REMediation (INCREM) that includes a combination of a Functional Remediation (FR) strategy plus a Computerized Cognitive Training (CCT) in order to improve not only cognitive performance but also the psychosocial functioning and the quality of life.

**Methods:**

A single blind randomized controlled clinical trial in 81 patients with a diagnosis of MDD in clinical remission or in partial remission. Participants will be randomized to one of three conditions: INCREM (FR + CCT), Psychoeducation plus online games and Treatment As Usual (TAU). Intervention will consist in 12 group sessions, of approximately 110 min once a week. The primary outcome measure will be % of change in psychosocial functioning after treatment measured by the Functional Assessment Short Test (FAST); additionally, number of sick leaves and daily activities will also be recorded as pragmatic outcomes.

**Discussion:**

To our knowledge, this is the first randomized controlled clinical trial using a combination of two different approaches (FR + CCT) to treat the present cognitive deficits and to promote their improvements into a better psychosocial functioning.

**Trial registration:**

Clinical Trials NCT03624621. Date registered 10th of August 2018 and last updated 24th August 2018.

## Background

Cognitive dysfunction is considered a new treatment target to improve psychosocial functioning and enhance the quality of life in patients with Major Depressive Disorder (MDD) [1, 2]. Previous research suggests that cognitive symptoms explain the low rates of global recovery and functional disability [3–5]. In fact, current pharmacological treatments for MDD have not been considered useful for the improvement of cognitive dysfunction probably because they are focused on improving mood [6]. The only antidepressants that seem to have a potential procognitive impact are duloxetine and vortioxetine [7–9], but further studies are needed to clarify these findings.

Regarding non-pharmacological treatments, Cognitive Remediation (CR) is a psychotherapeutic approach that has shown improvement in cognition in other neuropsychiatric disorders [10, 11] However, only few studies have been conducted in MDD patients [12–15]. The design features of the interventions differ from one another, making it difficult to demonstrate their efficacy. Moreover, samples included in CR studies were very heterogeneous with different disease burden, which would have impeded conclusions about the specific effect of CR for MDD. Analyses were also limited by the fact that not all patients show the same profile of cognitive impairment. It is estimated that 50% of depressed patients [16, 17] present enduring cognitive deficits, which would significantly interfere in workplace and in psychosocial functioning, while another large percentage of patients do not suffer from cognitive dysfunction.

Apart from that, most of CR programs have been based on neurological models designed to reverse cognitive deficits acquired after or associated with neurological conditions [18, 19], while the focus of CR programs in psychiatric diseases should be more directed in improving everyday functioning rather than merely recovering cognitive losses. Computerized Cognitive Training (CCT), which is based on cognitive exercises and games, appears to be, by contrast, an optimal method to improve cognitive functioning in affective disorders due to the flexibility given to adjust the tasks to the needs of each patient. In addition, and according to a recent meta-analysis [20], CCT improved depressive symptomatology and everyday functioning in patients with MDD, though producing inconsistent effects on cognition.

The scarce evidence on the efficacy of cognitive interventions may have prevented a broad implementation of such programs in the clinical setting. Moreover, research should define how cognitive remediation programs are to be administered (e.g., number of sessions), what cognitive domains are to be trained, and how this intervention can impact on psychosocial functioning. Therefore, there is a strong need of randomized clinical trials so as to demonstrate the efficacy of such interventions [14, 15].

Recently, one new ‘ecological’ intervention named Functional Remediation (FR) has been designed with the aim of transferring cognitive improvements to the daily functioning, by using neurocognitive techniques and training but also through psychoeducation on cognition and problem-solving. The FR program includes modeling techniques, role-playing, verbal instructions, self-instructions, positive reinforcement and meta-cognitive cues using real-life problems [21]. FR in bipolar disorder has shown significant enhancement of functional outcomes as well as subsyndromal symptomatology [22–24]. This is a group intervention and, thus, it is difficult to tailor the tasks to the specific needs of each individual.

Considering the above, the evidence accumulated indicates that the most adequate intervention to treat cognitive symptoms in MDD would be a combination of a group FR plus a personalized CCT. The inclusion of both aspects will allow the intervention to focus on the present deficits through the formation of new strategies with compensatory techniques and promoting their use into everyday life.

## Aims of the study

This study aims to show the efficacy of an INtegral Cognitive REMediation (INCREM) program specifically-designed for MDD patients so as to improve psychosocial functioning through the treatment of cognitive symptoms.

Primary research aims:

- To develop and to prove the efficacy of INCREM; this includes a FR program and a CCT.
- To improve psychosocial functioning in MDD patients.
- To decrease the rate of patients with enduring cognitive symptoms, and to reduce the risk of further relapses.

Secondary research aims:

- To establish cognitive profiles in MDD patients, to tailor the Integral Cognitive Remediation therapy so as to achieve full remission.
- To increase the effect size of the intervention by combining traditional group sessions plus computerized individual sessions.
- To study the specific elements (cognitive and psychosocial aspects) that mediate clinical and functional improvement, which can be ascertain through well-being and decrements in cognitive complaints.

## Methods and Design

This is a single blind, randomized controlled clinical trial approved by the Research Ethics Board of the Hospital de la Santa Creu i Sant Pau. All participants will receive extended information about the study and must give their written informed consent prior to the inclusion. The study will be carried out in accordance with the ethical principles of the declaration of Helsinki and Good Clinical Practices, complying with data protection laws with the anonymization of all the information collected (Data Protection Act 2018).

### Participants

Patients aged between 18 to 60 years with a diagnosis of MDD (DSM-5 criteria) will be recruited from the psychiatry department of the Hospital de la Santa Creu i Sant Pau in Barcelona (Catalonia, Spain) to participate in the present study. This center covers a population of 400.000 inhabitants, and therefore, the recruitment will be achieved. All patients will have to be in clinical remission or in partial remission, defined by scores below 14 in the HDRS-17. To be allocated in one of the treatment arms, patients will have to display objective cognitive deficits (measured with the Screening for Cognitive Impairment in Psychiatry – SCIP, defined by a score below 80) [25, 26] together with psychosocial dysfunction, defined by a score from 12 to 20 (mildly impaired) in the FAST [27]. All patients will continue with their usual pharmacological treatment. Exclusion criteria are: i) an intelligence quotient (IQ) below 85, ii) any medical condition that may affect neuropsychological performance, iii) presence of any comorbid psychiatric condition including non-nicotine substance use disorders on the previous three months, iv) having received electroconvulsive therapy on the previous year or psychological intervention in the previous 6 months.

### Study design

**Fig. 1:**
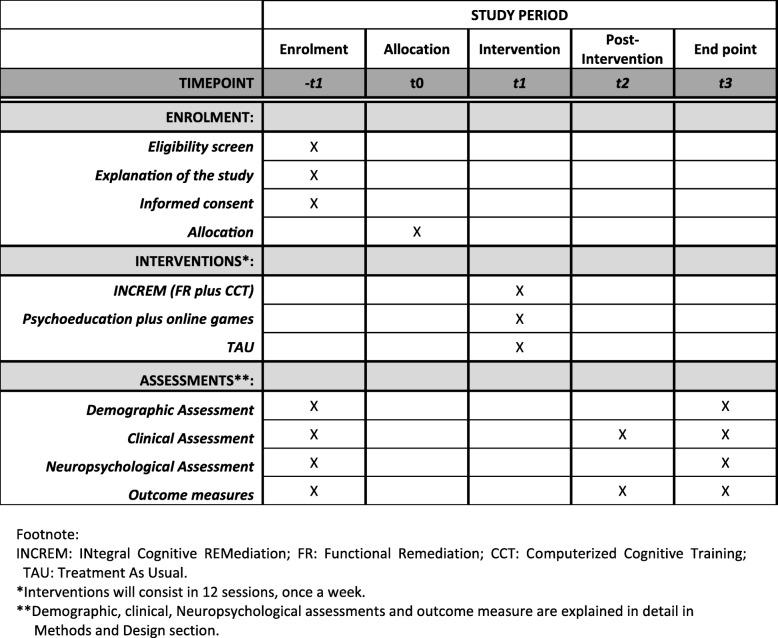
Schedule of enrolment, interventions, and assessments

Figure 1 provides a schedule of the study. Detailed oral and written information about the study will be provided to all potential candidates to participate in the study. If they are interested in participate, informed consent will be signed and demographic, clinical, neuropsychological and functional assessments will be performed by an experienced clinical neuropsychologist. After the fulfillment of the inclusion criteria, patients will be randomized to one of the three possible treatment options: INCREM program which includes both a FR program adapted to depression, and a CCT; Psychoeducation plus online mental skill games; and Treatment as Usual (TAU). A more detailed explanation of the two active treatment arms is provided below. An independent statistician from the Department of Epidemiology (Hospital de la Santa Creu i Sant Pau) will carry out the randomization by means of computer-generated random numbers. In order to ensure balanced sample sizes across groups over time, a block randomization method will be used. The block size will be set to 27, so as to have 9 patients per treatment arm, in 3 consecutive blocks. Blocks will then be randomly chosen to determine patients’ final assignment. Another experienced clinical researcher will assign participants to the corresponding intervention. The psychologists conducting treatment interventions will be different from the clinical neuropsychologist who will carry out the assessments and who will be blind to the treatment assignment. The intervention will be discontinued in case of request by the participant. Any other concomitant psychological treatment will not be permitted during the trial. Finally, to ensure adherence to intervention, after one unjustified absence rated by the psychologist, a phone call will be done prior to the next session.

After 3 months of treatment (12 sessions) efficacy measures will be collected: Functioning Assessment Short Test (FAST) [28], Perceived Deficit Questionnaire (PDQ-20) [29, 30], Hamilton Depression Rating Scale (HDRS-17) [31, 32], Remission from Depression Questionnaire (RDQ) [33] and 36-Item Short Form Health Survey, Version 2 (SF-36-V2) [34]. Then, 6 months after the intervention, a complete demographic, clinical, neuropsychological and functional assessment will be performed in order to evaluate the eventual long term effects. To promote retention and interest in the study by the participants, a detailed report about their evolution will be provided to them.

Estimation of sample size was set at 27 participants in each treatment arm, assuming a minimum difference on 6 points on the FAST (primary outcome), and considering a bilateral significance level of 5% and a statistical power of 80%, adjusted by a 20% rate of dropouts.

### Demographic and clinical assessment

A semi-structured interview will be used to assess demographic and clinical variables at baseline. Demographic assessment will include gender, age, years of education, age at which schooling was completed, work situation, work adjustment, marital status, cohabiting characteristics, and the number of children if any.

The Cognitive Reserve Assessment Scale in Health (CRASH; in press) will be included so as to estimate the cognitive reserve. It is a semi-structured interview-based scale that evaluates three domains considered fundamental in the construct of cognitive reserve: education, occupation and intellectual and leisure activities.

The clinical evaluation will cover age at illness onset, number of previous episodes, age of first psychiatric hospitalization, number of hospitalizations, period of clinical stability, seasonal pattern, presence of rapid cycling depression, presence of melancholy, atypical depression and psychotic symptomatology during depression, comorbidities in axis I, II and III, life history of suicidal ideation, life history of suicide attempts, number of attempts, method and medical severity used, and family history of psychiatric disorders and suicide. The HDRS-17 will be used to evaluate current depressive symptoms and assess clinical changes. In addition, the RDQ will be used to evaluate the patient-perceived remission that includes subscales for symptoms of depression, other symptoms such as anxiety and irritability, coping ability, positive mental health, functioning, life satisfaction and a general sense of well-being. These two scales will be analyzed as secondary clinical outcomes to evaluate the possible lasting effects.

### Neuropsychological assessment

Following the clinical assessment, a neuropsychological battery of tests will be administered by experienced neuropsychologists at the beginning of the study and at 12 months after intervention. It comprises standardized tests to assess attention, working memory, verbal and visual memory, processing speed and executive function. Premorbid Intelligence will be estimated with Vocabulary and Block Design subtests of the Wechsler Adult Intelligence Scale version-IV (WAIS-IV) [35]. Attention and verbal working memory performance will be evaluated with Digit forward and backward (WAIS-IV). The Conners Continuous Performance Test 3rd Edition (CPT-III) [36] will be used to assess sustained attention. Verbal learning memory will be measured by the California Verbal Learning Test (CVLT) [37]. Copy and recall of the Rey-Osterrieth Complex Figure (ROCF) [38] will be used to evaluate visuoconstructive abilities and visual memory. Processing speed will be measured by the Digit Symbol Substitution Test (DSST; WAIS-IV) and the Trail Making Test Part A (TMT-A) [39]. Finally, executive functioning will be measured with the Trail Making Test Part B (TMT-B) [39], the Stroop Color and Word Test (STROOP) [40], the Wisconsin Card Sorting Test (WCST) [41], the Tower of London (TOL) [42] and the Category Fluency and PMR (adapted for Spanish speaking population) [43, 44]. TMT-B is used to evaluate set-shifting abilities, STROOP interference is used to evaluate difficulties with response inhibition, WCST assess abstract reasoning ability and the ability to shift cognitive strategies in response to changing environmental contingencies, TOL is used to detect deficits in planning and problem-solving and Category Fluency and PMR are used to measure semantic and phonemic verbal fluency, respectively.

### Intervention

Selected patients are going to be randomized to three possible intervention arms: INCREM; PSYCHOEDUCATION + online games; and Treatment as Usual (TAU).

**INCREM** will consist of 12 sessions, of approximately 110 min once a week, involving a FR and a CCT. FR program was originally developed for bipolar patients [45] and it will be adapted for depressed patients creating a more intensive program focused on executive functions, memory and attention. The sessions aim at improving daily functioning based on ecological tasks where they need to use compensatory techniques. The specific explanation of the sessions is developed in Table 1. CCT will be applied right after FR group sessions in individual computer sessions during 20 min. The modules of training will be facilitated by the licensed game-like software CogniFit (https://www.cognifit.com). This neurocognitive stimulation program consists of a battery of tasks that allows depressed patients to improve their cognitive skills depending on their individual performance throughout different activities.

**Table 1:** Content of the 12-week Functional Rehabilitation Program

Session	Topics Covered
1. What is attention? (I)	Strategies of concentration
2. What is attention? (II)	Strategies to improve attention
3. Reading	Recovering the habit of reading
4. What is memory? (I)	Mnemonic strategies
5. What is memory? (II)	The use of diaries and external aids
6. What are executive functions? (I)	Self-instructions
7. What are executive functions? (II)	Programing activities, prioritization and time management
8. What are executive functions? (III)	Problem solving strategies
9. What are executive functions? (IV)	Working memory strategies
10. Stress	Stress and neurocognition
11. Communication	Communication skills and assertiveness
12. Autonomy	Independence and autonomy

**PSYCHOEDUCATION** will consist of 12 sessions, 90 min once a week involving psychoeducation sessions plus 20 min online non-directed game playing. This treatment arm has been designed as the control intervention arm due to its beneficial effects for depression. The material for the psychoeducation intervention has been specifically designed for the study and is based on validated treatment protocols [46–48]. Information about the disease is provided during the sessions so as to gain knowledge and strategies to cope with their own difficulties and to improve their quality of life. Therefore, the intervention will be divided in two modules: the first module (sessions 1 to 4) will consist in providing information about the disease and its treatment, and the second one (sessions 5 to 12) will consist of introducing different cognitive-behavioral techniques that have proven to be effective for depression. The specific explanation of the sessions is developed in Table 2. The online game will be applied right after the psychoeducation sessions during 20 min. The games will be facilitated by a free mini-game online website (www.frivplus.com). No records will be gathered on this last part, as it is only used to make comparable the two active treatment arms.

**Table 2:** Content of the 12-week Psychoeducational Program

Session	Topics Covered
1. What is depression? (I)	Concept of depression
2. What is depression? (II)	Definition, symptoms, prevalence and course
3. Etiology of depression	Biopsychosocial model, psychobiology and psychological theories
4. Treatment of depression	Pharmacological, psychological and other treatment options
5. Behavioral Activation (I)	Introduction to Behavioral Activation
6. Behavioral Activation (II)	Exposition to positive activities
7. Cognitive Distortions	Cognitive distortions, automatic negative thoughts and cognitive restructuring technique
8. Anxiety	Anxiety management strategies
9. Social Skills (I)	Social skills deficits and communicational styles
10. Social Skills (II)	Social skills training
11. Problem-Solving Technique	Problem-Solving training
12. Relapse Prevention	Summary of the sessions and relapse prevention

**TAU** will consist in giving the usual treatment to patients according to accepted standards for depression (except psychotherapy which will not be allowed 6 months before the study nor during it).

### Outcome measures

The primary outcome measure of the study will be the change in psychosocial functioning after treatment, which will be measured by means of the FAST. The FAST is a scale created to assess disability in functioning in mental health which contains 24 items evaluating autonomy, occupational functioning, cognitive functioning, financial issues, interpersonal relationships and leisure time. The scores range from 0 to 72, with higher scores indicating a greater disability. A sum of ≥12 represents a mildly to severe functional impairment in patients with bipolar disorder [27]. Given the complexity of the possible effects of the intervention, changes in pragmatic variables of psychosocial functioning will also be evaluated: number of sick leaves and number of daily activities.

Different secondary outcome measures will be analyzed: i) the change in cognitive performance across cognitive domains (through composite scores of the different cognitive domains and a global composite cognitive score); ii) change in subjective appraisal of cognitive functioning evaluated with the PDQ-20 which is a self-reported questionnaire that measures the patients’ perception of their cognitive functioning. It consists of 20 items assessing attention, retrospective memory, prospective memory, and planning and organization; iii) change in depressive symptoms with the HDRS-17); iv) change in patients’ perspective of clinical remission with the RDQ; and v) change in quality of life assessed with the SF-36-V2. It evaluates the patients’ health-related quality of life gauging eight sections: vitality, psychical functioning, bodily pain, general health perceptions, physical role functioning, emotional role functioning, social role functioning and mental health.

### Statistical analysis plan

Data will be collected in three different moments: baseline, post-intervention and follow-up through a logbook data collection. Then, an online application called Clinapsis will be used for data collection, which ensures anonymisation of data (through encoding system). This system will grant public access to the full protocol and dataset at participant-level for further analysis beyond this proposal.

The data will be analyzed using the Statistical Package of Social Sciences (SPSS, IL, Chicago, version 20) and R Package (version 3.1.2). First, baseline differences between groups will be investigated using chi-square tests and ANOVAs depending on the nature of the variables. Secondly, mixed-models will be carried out to assess the impact of the three different treatment options on the psychosocial functioning scores assessed with the FAST scale (primary outcome measure). Effect sizes of interventions will be calculated. Secondary variables will also be analyzed with the same statistical approach. Mixed repeated measures ANOVAs will be carried out to investigate the long-term effects of interventions on secondary outcomes. Finally, in order to investigate predictors of treatment response, a study of mediators and moderators will be conducted. For the main statistical analysis, the Last Observation Carried Forward method will be used to minimize the effect of dropout rates at 6 months of follow-up after intervention.

## Discussion

Cognitive impairment is nowadays considered a core symptom of MDD and a critical determinant of psychosocial and workplace outcomes, as well as health outcomes such as the health-related quality of life. CR has been used to treat these cognitive deficits suggesting significant improvements in cognition but with small effect sizes for psychosocial functioning. This study aims to improve psychosocial functioning through the improvement in cognitive performance. To our knowledge, this is the first randomized clinical trial using an INtegral Cognitive REMediation (INCREM) composed by 12 sessions of a FR program and a CCT.

The major strength of this study is the combination of two different approaches at the same time: a group format therapy together with a computerized program that allows a personalized delivery of the intervention. This synergistic approach may represent the path to optimize treatment response and ensure better functional outcomes in patients with MDD. The presence of an active control group beyond treatment as usual (TAU) is another relevant strength of the study. A large battery of primary and secondary outcome measures will be used to evaluate the efficacy of the intervention. Patients will be approached six months after finishing the intervention to assess the maintenance of possible changes. Furthermore, this study will take into account the specific cognitive deficits of each patient by adapting CCT to individual performance.

A potential limitation of the present study is inherent to any clinical trial, given that participation and follow-up of the interventions could be more complicated. A 20% of dropouts are calculated throughout the intervention and/or follow-up has been estimated, but this percentage could be higher, which would limit the power of the analysis. Patients should be in remission or in partial remission with cognitive deficits and difficulties in daily life, so that recruitment and inclusion might be more complicated. Concomitant medication might also represent a possible limitation because of side effects or even procognitive effects of newer drugs. Medication regimes will be recorded during the different phases of the study and then will be taken into account in the analyses. In any case, in the clinical practice, polymedicated patients are the rule rather than the exception. Hence, it might limit internal validity but it is strength for the generalization of the results.

## Conclusion

The presence of residual cognitive symptoms, observed in the clinical settings, may impede the achievement of full remission in patients with depression. Therefore, effective cognitive treatments are an unmet need. If the results of this study are conclusive, the INCREM could be added to the therapeutic arsenal for depression. This non-pharmacological treatment may seem expensive as it consists in 12 sessions, but if one considers the direct and indirect social and healthcare costs of depression [49], then targeting cognitive symptoms with a cognitive program will likely be cost-effective.

## Abbreviations

CCT: Computerized Cognitive Training
CPT-III: Conners Continuous Performance Test 3rd Edition
CR: Cognitive Remediation
CRASH: Cognitive Reserve Assessment Scale in Health
CVLT: California Verbal Learning Test
DSST: Digit Symbol Substitution Test
FAST: Functioning Assessment Short Test
FR: Functional Remediation
HDRS-17: Hamilton Depression Rating Scale
INCREM: INtegral Cognitive REMediation
IQ: Intelligence Quotient
MDD: Major Depressive Disorder
PDQ: Perceived Deficit Questionnaire
RDQ: Remission from Depression Questionnaire
ROCF: Rey-Osterrieth Complex Figure
SCIP: Screening for Cognitive Impairment in Psychiatry
SF-36-V2: 36-Item Short Form Health Survey, Version 2
STROOP: Stroop Color and Word Test
TAU: Treatment As Usual
TMT-A: Trail Making Test Part A
TMT-B: Trail Making Test Part B
TOL: Tower of London
WAIS-IV: Wechsler Adult Intelligence Scale version-IV
WCST: Wisconsin Card Sorting Test
